# Complaints and Diagnoses of Emergency Department Patients in the Netherlands: A Comparative Study of Integrated Primary and Emergency Care

**DOI:** 10.1371/journal.pone.0129739

**Published:** 2015-07-01

**Authors:** Wendy A. M. H. Thijssen, Elske van Miero, Maartje Willekens, Jasper Rebel, Maro H. Sandel, Paul Giesen, Michel Wensing

**Affiliations:** 1 Emergency Department, Catharina Hospital, Eindhoven, the Netherlands; 2 IQ Scientific Institute for Quality of Health Care, Radboud University, Nijmegen Medical Centre, Nijmegen, the Netherlands; 3 Emergency Department, Canisius Wilhelmina Hospital, Nijmegen, the Netherlands; 4 Emergency Department, Onze Lieve Vrouwe Gasthuis, Amsterdam, the Netherlands; 5 Emergency Department, Haga Hospital, The Hague, the Netherlands; Robert Bosch Hospital, GERMANY

## Abstract

**Objective:**

In the Netherlands, an increasing number of emergency departments (EDs) and general practitioner cooperatives collaborate by creating one Emergency-Care-Access-Point (ECAP). This has resulted in fewer patients at ECAP EDs. The objective of this study was to explore differences in patient characteristics, presented complaints and ED discharge diagnoses between EDs with an ECAP and EDs without an ECAP.

**Methods:**

A retrospective observational study was performed with 1800 consecutive patient records sampled from six EDs spread over the Netherlands in 2013. We extracted data on time and date of presentation, sex, age, presenting complaint, discharge diagnosis, origin and follow up.

**Results:**

At ECAP EDs, the mean age was 47.8 years (95%CI 46.1-49.4) compared to 41.3 (95%CI 39.7-42.9). Compared to non-ECAP EDs, more patients were referred by medical professionals (74.7% versus 46.8%), more patients received hospital admission (45.2% versus 29.0%) and fewer patients received GP follow-up (4.1% versus 16.9%). There was no significant difference in presenting complaints between ECAP and non-ECAP EDs. Most prevalent complaints were trauma (25.7% versus 29.7%), abdominal pain (12.1% versus 10.9%) and general symptoms (7.8% versus 4.8%). The most prevalent ED diagnoses significantly differed with fractures and dislocations (10.8%), sprains and strains (10.4%) and respiratory infections (6.8%) at ECAP EDs versus fractures and dislocations (10.7%), wounds (9.3%) and sprains and strains (8.9%) at non-ECAP EDs.

**Conclusion:**

Compared to non-ECAP EDs, patients at ECAP EDs were older, medical professionals referred more patients and more patients received a hospital admission. We found some small differences in discharge diagnoses between ECAP EDs compared to non-ECAP EDs, but no difference in presented complaints.

## Introduction

### Background

Rising healthcare costs and crowding of emergency departments have worldwide led to initiatives to redirect the self-referred patient to the general practitioner (GP),[1–5] Readily available access to the GP, especially after hours is a precondition for this. In contrast to the 29–40.2% of GPs providing after-hours care in the United States, after-hours care is provided by nearly all GPs in several countries, including the Netherlands, New Zealand and the UK.[6,7] Emergency care in the Netherlands is mainly provided by emergency departments (EDs), ambulance services and GPs. In the last 15 years GPs have changed their after-hours care from small rota-groups into large GP-cooperatives (GPCs). There are now more than 130 GPCs serving more than 90% of the Dutch population,[8] A GPC is open from 5 pm to 8 am daily, the entire weekend and on public holidays. Patients seeking after-hours care can phone a single, regional telephone number where assistants perform telephone triage and either book a consultation at the GPC, send a GP on a home visit or give a self-care advice,[9] All phone calls are authorised by a GP. Over 90% of the GPCs use the Netherlands Triage Standard (NTS), a system that not only decides who needs to be treated first but also who can be treated by a GP or needs to be referred to an ED. The GPCs handle the majority of all after-hours patient contacts (88%) and refer around 5–7% to the ED.[10,11] Despite the implementation of a GPC, patients can still bypass the GP and visit the ED at their own initiative, since they are situated in different locations. To redirect these self-referred ED patients to the GP, an increasing number of emergency departments and GPCs collaborate by creating one Emergency Care Access Point (ECAP). At an ECAP patients are initially triaged under the supervision of a GP. Depending on the outcome, the patient is then treated at the GPC or referred to the ED. With an ECAP, patients no longer have to decide whether their complaint needs to be treated at an ED or by a GP, since they have one desk where they can seek care. Depending on the hospital, patients that are seen in the ED, can be triaged with different systems like the Manchester Triage System (MTS), Emergency Severity Index (ESI) or the Netherlands Triage Standard (NTS).

A long-term pre-post follow-up study found that the introduction of an ECAP led to a 27% decrease of ED patients after-hours and a 30% increase in GPC contacts. This was mostly due to a shift of the low acuity self-referral and changed both admission rates and acuity of patients presenting at an ECAP emergency department,[12] Several single centre pilot studies, with short follow-up periods, also found a shift of low acuity patients, mostly self-referrals, from the ED to the GPC, varying from 15% to 53%. After implementation of an ECAP, the patient at an ECAP ED is older and more referred compared to the pre-ECAP situation,[4,13–19]. This change in patient flows, might explain the change in relative increase of hospital admissions at ECAP EDs, but not the absolute increase that was found as well.[12] Despite previous research, it is currently unknown why the presence of an ECAP leads to an increased admission rate in the ED. Potential reasons for this increase include an increase in adherence with more GP referrals, unwillingly creating an induced demand system or a lower threshold for admission of referred patients than for self-referred patients for similar complaints. We wondered whether a GPC at an ECAP, refers patients with specific complaints to the ED more often than GPCs that do not have an ECAP or that a GPC refers the sicker patients of every category of patients to the ED. In the latter situation, the type of complaints seen in the ED is equal, yet they are more likely to represent a sicker patient group or patients with a higher risk-stratification.

### Importance

Pre-post studies showed that ECAP EDs see older mostly referred patients with higher absolute admission rates after implementing an ECAP [4,13–19]. One factor associated with an increase in hospital admissions could be differences in presented complaints and ED discharge diagnosis between EDs with and EDs without an ECAP. This has never been studied comparing multiple ECAP EDs and non-ECAP EDs.

### Aim

This study aimed to provide insight into the clinical characteristics of patients presenting at ECAP EDs compared to non-ECAP EDs. It will particularly focus on the presented complaints and ED discharge diagnoses, where we expect differences between the two types of organisations.

## Methods

### Design and study population

We performed a retrospective observational study of patients sampled from ED records of six emergency departments spread over the Netherlands, varying from urban EDs to large inner city EDs. The nationally recognised medical ethical committee of the Catharina Hospital, the Netherlands granted institutional review board exemption. All patient records were made anonymous and de-identified prior to analysis. Since there are only a few ECAP EDs, we conveniently selected three EDs that were integrated with GP cooperatives forming one Emergency Care Access Point (ECAP) and three comparable EDs in size and location (urban or inner city) that were not integrated and made sure that they were evenly spread over the country. Table 1 describes the characteristics of the participating hospitals and EDs, which were evenly spread over the country, comparable in size and included the two largest national EDs, one with an ECAP and one without an ECAP.

**Table 1:** Overview of hospital and ED characteristics divided in ECAP and non-ECAP hospitals over 2012.

		Overview Hospitals		
		ECAP hospitals	Non-ECAP hospitals	Total
**Hospital**	**Urbanization**	2 Urban, 1 Innercity	2 Urban, 1 Innercity	—
	Total hospital beds*	654–696	545–955#	545–955#
	Total admissions*	99.412	79.763	179.175
	ED admissions of total hospital admissions (%)*	32.7	37.5	34.8
	Mean length hospital stay (days) *	4.3–4.8^	5.0–5.4^	4.3–5.4
**ED**	Total ED patients per year*	99.859	113.933	213.792
	Total ED admissions*	32.506	29.883	62.389
	ED admissions of total ED presentations (%)*	32.6	26.2	29.2
	**EP present 24/7@**	1 Yes, 2 No	2 Yes, 1 No	—
	**Triage system<**	2 MTS, 1 NTS	1 MTS, 2 ESI	—

^ N/A in year report, calculated (Known admissions and length of hospital stay).

* table presenting numbers of the year 2012 both office and ECAP hours.

# also includes daycare beds.

@ when EPs are not present 24/7 they are not there during night hours.

<MTS Manchester Triage System, NTS Netherlands Triage System, ESI, Emergency Severity Index.

In the ECAP group, two hospitals had an emergency cardiac care unit, one hospital was a tertiary cardiac referral centre performing primary cardiac interventions and one hospital was a dialysis centre. This was the same in the non-ECAP group. GPCs at two ECAPs could order X-rays during evening hours without referring the patient to the ED, one could not. This was not possible at night for all of them. One non-ECAP ED was also a trauma-centre. At an ECAP, patients have one access point and triage decides whether the patient is seen by a GP or in the ED. At EDs without an ECAP, patients can bypass the GP and present directly to the ED. We collected a total of 1800 patient records, 300 patients attending each ED after triage, starting 1 February 2013. To make sure we only included patients during ECAP opening hours (out-of hours), we collected the first consecutive 100 patient visits during the evening, the first 100 at night and the first 100 during weekend days, a total of 300 patients of each hospital. This could comprise several days.

### Measures

All participating EDs use computerised charts, which were used for sampling. Using a standardised registration form we collected time and date of presentation, sex, age, acuity, presented complaint, discharge diagnosis, origin (self-referred, referred by medical professionals) and follow-up (admission, follow-up at the out-patient clinic or GP, no follow-up). Patients who were registered in the system, but did not receive healthcare in the ED were not included. For instance we did not include a patient who after registration, was seen directly in the obstetric ward. Some patients referred by the GP could arrive by ambulance. Depending on the hospital registration system, these patients were either coded as “referred by GP” or “arrived by ambulance”. We combined the two options in one category: “referred by medical professionals. This category also included private 112 calls for an ambulance, which theoretically are self-referred patients. Ambulance paramedics in the Netherlands, however, are not obligated to transfer every 112-calling patient to the ED, instead they can contact the patients GP for a consult or request the patient to use other means of transportation. We therefore believe that the percentage of low-acuity self-referrals that should not have been transported to the ED by ambulance is small in the “referred by medical professionals” group. We first coded the presented complaint according to the International Classification of Primary Care 2^nd^ edition of the Wonca International Classification Committee (ICPC-2-NL) and the single best discharge diagnosis using the ICD-10 version 2006 of the Dutch WHO-FIC Collaborating Centre. We then grouped similar ICPC codes and ICD 10 codes for analysis purposes and named them post-hoc groups. For instance, in the ICPC codes we grouped abdominal pain, which contains D01 (Generalised abdominal pain), D02 (Stomach pain) and D06 (Other localised abdominal pain). For the ICD 10 codes, for example, we grouped chronic respiratory disease, which contains J44.1/9 (COPD), J45.9 (Asthma) and J 46 (Status asthmatics) or Acute coronary syndrome, which contains I21.9 (Myocardial infarction), I20.0/9 (Unstable angina) and I22 (Subsequent myocardial infarction). We collected descriptive data specific to each hospital and ED, which could have an effect on patient characteristics, like tertiary referral cardiac hospital or dialysis, triage system and annual patient ED visits in 2012. (Table 1). Since hospitals do not routinely code in ICPC or ICD-10, two researchers (WT and EvM) independently coded all presented complaints on the basis of recorded information. When there was a difference in coding for both ICPC and ICD-10, mutual agreement was reached through following a pre-set protocol. (S1 Appendix ICPC and S2 Appendix ICD10 respectively) This led to consensus in all cases. ICD-10 coding was based on the diagnosis on the discharge notes in the ED or when admitted and available, on the discharge letter from the admitting specialty. When no final diagnosis was recorded the presenting complaint was coded as an ICD-10 code.

### Outcomes

The primary outcomes of interest in this study were patients’ presented complaint and ED discharge diagnosis. Secondary outcomes were patients’ origin (self-referred, referred by medical professionals) and follow-up (admission, follow-up at the out-patient clinic or PCP, no follow-up).

### Analysis

Integrity was checked for all data and entered in a research database. We used IBM SPSS version 19 for statistical analysis. To compare differences between ECAP EDs and non-ECAP EDs, descriptive statistics were used. We analysed for differences with a T-test or chi-square test where appropriate. We considered P < 0.05 statistically significant. We explored whether patients’ presented complaint and discharged diagnosis was related to the presence of an ECAP. We grouped similar complaints and diagnosis post-hoc to reach at least a minimum of 80% of cells expected count more than five.

## Results

In 2012, a total of 213.792 patients attended the researched EDs of which 62.389 patients were admitted to hospital. This represents an admission rate of 29.2% of all ED attendances and 34.8% of all hospital admissions. All hospital EDs were open 24/7, 3 of which had emergency consults on at night. (Table 1)

Table 2 shows that of the total of 1800 included patients, 54.7% was male and 30.8% presented with a trauma related problem. The mean age was 45 years old (median 44.0) with the majority of patients aged between 31 and 50 years old. Of all ED attendances, 29.9% were self-referred, medical professionals referred 60.7% and 3.4% were referred by the radiology department. A total of 37.1% required a hospital admission, 25.9% were followed up at the out-patient clinic, 10.5% were followed up by their own PCP during office hours and 22.2% did not require any follow up.

**Table 2:** Patient characteristics and care pathways, comparing ECAP and non-ECAP EDs (n = 1800).

		ECAP ED	Non-ECAP	Total
**Male(%), P = 0.570^**		54.0	55.4	54.7
**Trauma (%) N = 1793, P = 0.086^**		28.8	33.3	30.8
Age (%), P<0.01*	**Median (years)**	50.0	39.0	44.0
	**0–5**	7.1	7.4	7.3
	**6–18**	7.9	11.8	9.8
	**19–30**	15.8	21.1	18.4
	**31–50**	20.3	22.9	21.6
	**51–65**	18.9	16.4	17.7
	**66–85**	25.2	16.3	20.8
	**>85**	4.8	4.0	4.4
**Origin (%)#, P<0.01^**	**Self-referrals**	15.9	44.0	29.9
	**Referred by medical professionals**	74.4	46.8	60.7
	**Referred by the radiology department**	5.3	1.2	3.4
**Follow-up (%)#, P<0.01^**	**Hospital admission**	45.2	29.0	37.1
	**Out patient clinic**	26.8	25.1	25.9
	**PCP Follw-up**	4.1	16.9	10.5
	**No follow-up**	19.7	24.7	22.2

^#^ Does not add up to 100% due to other options not shown in the table.

^+^ Includes ambulance and PCP referred patients.

* independent T-test.

^ Chi square test.

### ECAP versus non-ECAP

Table 2 also presents the descriptive patient data of ECAP and non-ECAP EDs. At ECAP EDs the average patient age was 47.8 years (median 50.0) compared to 41.3 years (median 39.0) for non-ECAP EDs. ECAP EDs treated 15.9% self-referred patients who presented at the ECAP and, after triage were seen at the ED. Non-ECAP EDs treated 44.0% self-referred patients who were seen at the ED directly. At ECAP EDs, more patients were referred by medical professionals (74.7% versus 46.8%) and more patients were referred by the radiology department (5.3% versus 1.2%). At ECAP EDs, more patients received a hospital admission (45.2% versus 29.0%) or a follow-up at an out-patient clinic (26.8% versus 25.1%) and fewer patients received a GP follow-up (4.1% versus 16.9%) or no follow-up (19.7% versus 24.7%).

### Presented complaint

Table 3 shows the percentage of presented complaints in each ICPC domain comparing ECAP and non-ECAP EDs. The three most prevalent categories in the ICPC domains were equally represented, namely category A, General and Unspecified (53.6% versus 52.2%) D, Digestive. (15.4% versus 16.0%) and R, Respiratory (9.9% versus 8.3%).

**Table 3:** ICPC codes for most presented complaint within each domain.

P value	ECAP EDs	N	%	Non-ECAP EDs	N	%
0.07	**1 General, Unspecified (A)**	482	53.6	**1 General, Unspecified (A)**	470	52.2
	**2 Digestive (D)**	139	15.4	**2 Digestive (D)**	144	16.0
	**3 Respiratory (R)**	89	9.9	**3 Respiratory (R)**	75	8.3
	**4 Process codes (St)**	43	4.8	**4 Neurological (N)**	39	4.3
	**5 Neurological (N)**	40	4.4	**5 Musculoskeletal (L)**	36	4.0
	**6 Musculoskeletal (L)**	28	3.1	**6 Process codes (St)**	30	3.3
	**7 Cardiovascular (K)**	22	2.4	**7 Skin (S)**	29	3.2
	**8 Urological (U)**	16	1.8	**8 Psychological (P)**	22	2.4
	**9 Psychological (P)**	13	1.4	**9 Urological (U)**	17	1.9
	**10 Skin (S)**	13	1.4	**10 Cardiovascular (K)**	14	1.6
	**Total**	900	%		900	%

Process codes (St) are *63 (return visit check-up) *62 (No letter available), *69 (problems with iv line or drain or healthcare workers needle stick injuries etc.

Within these three ICPC domains the most presented complaints are similar for ECAP and non-ECAP EDs, namely Trauma (47.9% versus 56.8%), Localised abdominal pain (38.8% versus 32.6%) and Shortness of breath (58.4% versus 57.3%). (Table 4)

**Table 4:** Most frequent presented ICPC domains.

ECAP EDs N = 900				Non-ECAP EDs N = 900			
ICPC domain, % of total	Most frequent presenting complaint within ICPC domain	N	% within ICPC domain	ICPC domain of total %	Most frequent presenting complaint within ICPC domain	N	% within ICPC domain
A General Unspecified, N = 482, 53.6%	Trauma (A80)	231	47.9	A General Unspecified, N = 470, 52.2%	Trauma (A80)	267	56.8
	General symptoms (A29)	69	14.3		General symptoms (A29)	43	9.1
	Chest pain (A11)	54	11.2		Worried of treatment (A13)	39	8.3
D Digestive, N = 139, 15.4%	Localized abdominal pain (D06)	54	38.8	D Digestive, N = 144, 16.0%	Localized abdominal pain (D06)	47	32.6
	Generalized abdominal pain (D01)	44	31.6		Generalized abdominal pain (D01)	42	29.2
	Stomach ache (D02)	11	7.9		Vomiting (D10)	14	9.7
R Respiratory, N = 89, 9.9%	Shortness of breath (R02)	52	58.4	R Respiratory, N = 75, 8.3%	Shortness of breath (R02)	43	57.3
	Coughing (R05)	22	24.7		Coughing (R05)	16	21.3
	Epistaxis (R06)	9	10.1		Epistaxis (R06)	4	5.3

After post-hoc grouping of the complaints into categories, the three categories with highest prevalence remained the same for ECAP and non ECAP EDs, namely, trauma (25.7% versus 29.7%), abdominal pain (12.1% versus 10.9%) and general symptoms (7.8% versus 4.8%). Overall, we found no significant differences in presented complaints between ECAP EDs and non-ECAP EDs.

### ED Discharge diagnosis

Table 5 shows that, when comparing discharge diagnosis between ECAP EDs and non-ECAP EDs, within the ICD 10 domains, the three most prevalent domains were equally represented: “Injury, poisoning and certain consequences of external causes” (30.8% versus 35.4%), “Symptoms, signs and abnormal clinical and laboratory findings, not elsewhere classified” (19.2% versus 19.2%) and “Diseases of the respiratory system” (10.9% versus 9.6%).

**Table 5:** ICD 10 Discharge diagnosis codes in each domain.

P value	ECAP EDs	N	%	Non-ECAP EDs	N	%
0.032	**XIX Injury, poisoning**	277	30.8	**XIX Injury, poisoning**	319	35.4
	**XVIII Symptoms, signs**	173	19.2	**XVIII Symptoms, signs**	173	19.2
	**X Respiratory**	98	10.9	**X Respiratory**	86	9.6
	**IX Circulatory**	66	7.3	**XX External causes**	54	6.0
	**XI Digestive system**	59	6.6	**XI Digestive system**	48	5.3
	**I Infectious**	37	4.1	**XXI Factors influencing health**	42	4.7
	**XX External causes**	37	4.1	**IX Circulatory**	39	4.3
	**XXI Factors influencing health**	33	3.7	**I Infectious**	32	3.6
	**XIV Genotourinary**	31	3.4	**XIV Genitourinary**	24	2.7
	**VI Nervous system**	19	2.1	**VI Nervous system**	18	2.0
	Total*	900			900	

Free text of the codes is shortened for table purposes.

* Does not add up to a total of 100% due to domains not presented in this table.

N = number of patients.

After post-hoc grouping, there was a significant difference in specific discharge diagnosis. The three categories with highest prevalence were Fractures and dislocations (10.8%), Sprains and strains (10.4%) and Respiratory infections (6.8%) for ECAP EDs compared to Fractures and dislocations (10.7%), Wounds (9.3%) and Sprains and strains (8.9%) for non ECAP EDs. (Table 6).

**Table 6:** Top ten of grouped ICD 10 codes^#^.

P value	ECAP EDs	N	%	Non-ECAP EDs	N	%
***P = 0*.*001***	Fractures and dislocations	97	10.8	Fractures and dislocations	96	10.7
	Sprains and Strains	94	10.4	Wounds	84	9.3
	Respiratory infections	61	6.8	Sprains and Strains	80	8.9
	Abdominal pain, unspecified	50	5.6	Abdominal pain, unspecified	56	6.2
	Specific abdominal diagnosis	44	4.9	Respiratory infections	55	6.1
	Neurological diseases	40	4.4	Other^	50	5.6
	Other^	40	4.4	Neurological diseases	35	3.9
	Chest pain, unspecified	39	4.3	Intoxications	33	3.7
	Urogenital diseases	34	3.8	Urogenital diseases	32	3.6
	Intoxications	32	3.6	Specific abdominal diagnosis	31	3.4
		900*	**%**		900*	**%**

# free text of the codes is shortened for table purposes.

*Does not add up to total 900, due to categories not shown in this table.

^ contains diagnoses not fitting in any other group and to small for a single group, (for example Glaucoma, allergic reaction).

N = number of patients.

For both ECAP and non-ECAP patients, in 15.0% and 14.4% respectively no diagnosis was made. Of this group the most recorded discharge diagnosis was Unspecified abdominal pain (5.6% versus 6.2%), Unspecified chest pain (4.3% versus 2.9%) and Syncope (1.1% versus 1.8%).

## Discussion

This study explored the influence of an ECAP on ED patient characteristics, complaints and discharge diagnoses. Compared to non-ECAP EDs, patients at ECAP EDs were older, more patients were referred by medical professionals and more patients received a hospital admission. We found some small differences in discharge diagnoses between ECAP EDs compared to non-ECAP EDs, but no difference in presented complaints.

Our study found a higher percentage of patients, referred to the ED at an ECAP ED (74,7%) compared to a non-ECAP ED (46,8%). This finding is similar to previous Dutch studies with pre-post ECAP settings, showing a shift of patients from secondary to primary care, resulting in a decrease of the number of ED patients and an increase of the number of GPC patients,[4,8,12]. Although, we used multiple ECAP EDs and non-ECAP EDs spread over the country, instead of a pre-post setting, the differences we found in patient characteristics and flows, especially a higher hospital admission at ECAP EDs compared to non-ECAP EDs are consistent with previous studies,[4,13–20] We found no differences between ECAP EDs and non-ECAP EDs for presented complaints. Our study showed that trauma was the most prevalent reason for consulting the ED. After trauma, in both ECAP and non-ECAP EDs the most presented complaints were abdominal pain and respiratory problems. These findings are supported by previous studies in Europe, although some studies found chest pain as most presented complaint,[14,21–24] In our research setting, most patients with chest pain, especially the referred patients are likely to have been seen directly at the emergency cardiac care unit, bypassing the ED and therefore comprise a small percentage of all patients, especially within the referred group. In an Asian study, the most prevalent complaint is respiratory problems, mostly asthma related,[24] In the United States, ED patients mostly present with stomach and abdominal pain, fever, cough, headache and back symptoms,[25,26] The latter four are complaints, patients in the Netherlands mostly present with at the GP,[27] The difference in ED patient characteristics with similar complaints, suggests that co-factors, like higher age and referral, are more predictive of admission then the actual complaint. Older and referred patients have a higher a-priory chance of a more serious disease and a higher likelihood of staying within the hospital setting after being referred,[20,28] Furthermore, single centre studies show that the low acuity referred patient has a higher admission rate and more diagnostic tests performed compared to the self-referring low-acuity patient,[12,20]

Our study found a significant difference in patient discharge diagnoses, mostly caused by wounds, unspecified chest pain and specific abdominal diagnosis, where we found more patients with wounds and fewer patients with unspecified chest pain and specific abdominal diagnosis at Non-ECAP EDs. The GPC deals with the majority of all after-hours patient contacts (88%) and refers around 5–7% to the ED,[9,10,27] The presentation of patients at an ECAP ED with similar complaints compared to non-ECAP EDs, yet a higher percentage of admissions and difference in discharge diagnoses, suggests an appropriate referral to the ED. For the ED, however it is unclear if all admitted patients require a hospital admission on the basis of their clinical presentation, as a referral alone has a higher likely hood of an admission,[20,28]

There is a growing tendency in recent years to intensify collaboration between primary-care and hospital-based emergency care. The ECAP EDs in the Netherlands represent the closest form of collaboration between primary care physicians and hospital emergency physicians. The main reason for implementing ECAPs is the reduction of healthcare costs by redirecting the low-acuity self-referred patient to the GP. With a resulted decrease in overall ED utilisation, this seems a success. However, since the number of referred patients increased and they have more diagnostic tests performed and are more likely to be admitted, this questions the actual reduction in healthcare costs. Insight is needed into the reasons for general practitioners to refer a patient to the ED (sicker patient, no GP possibility for ancillary testing, ED is just next door and thus convenient) and for ED physicians to admit a patient (medically needed, referred by the GP). This will optimise the ECAP setting, reduce healthcare costs and maintain quality of care.

### Limitations

Patient records do not routinely register ICPC and ICD-10 codes. This was done purposefully for this study by two trained clinicians. Since this was a retrospective analysis, coding was based on available information, which could have led to wrongful coding in some cases. This also counts for grouping the codes. Published studies did not clearly describe how this grouping was done; it is therefore difficult to compare outcomes. For analysis purposes, we grouped similar complaints and diagnosis post-hoc to reach at least a minimum of 80% of cells expected count more than five. This unfortunately, enlarges patient complaint and diagnoses group and will lead to a loss of specific diagnoses. We found no large differences in acuity between ECAP and non-ECAP EDs. Unfortunately, we could not statistically compare groups as different triage systems were used.

This study was done in the Netherlands, a country with a well-developed primary care system. Conclusions of this study might therefore not be applicable to countries where primary care is less well accessible or less comprehensive regarding ambulatory care-sensitive conditions (e.g. diabetes and COPD).

This study looked at the first complaint and diagnosis registered on the discharge note. There is a bias from the treating physician writing the progress notes.

### Conclusion

In summary this study showed that ED patients in the Netherlands present with similar complaints but at ECAP EDs, they seem more seriously ill or at risk, given their higher age, referral status and admission rates. Although we could not look at acuity, our results suggest that the implementation of an ECAP is an important factor, influencing emergency hospital care.

## Supporting Information

S1 Appendix Coding principles ICPC. (DOC)

S2 Appendix Coding principles ICD10. (DOCX)
