# Correction: Complaints and Diagnoses of Emergency Department Patients in the Netherlands: A Comparative Study of Integrated Primary and Emergency Care

**DOI:** 10.1371/journal.pone.0133947

**Published:** 2015-07-20

**Authors:** The PLOS ONE Staff

The second author’s name is spelled incorrectly. The correct name is: Elske van Mierlo. The correct citation is: Thijssen WAMH, van Mierlo E, Willekens M, Rebel J, Sandel MH, Giesen P, et al. (2015) Complaints and Diagnoses of Emergency Department Patients in the Netherlands: A Comparative Study of Integrated Primary and Emergency Care. PLoS ONE 10(7): e0129739. doi:10.1371/journal.pone.0129739. The publisher apologizes for the error.
