# Supplementary material for: Complaints and Diagnoses of Emergency Department Patients in the Netherlands: A Comparative Study of Integrated Primary and Emergency Care
Source: PLoS One. 2015 Jul 1;10(7):e0129739. doi: 10.1371/journal.pone.0129739 (PMC4488864; doi:10.1371/journal.pone.0129739)
Supplement: S1 Appendix — (DOC) [file pone.0129739.s001.doc]

**Appendix 1. Coding principles**

For coding complaints (not immediately obvious) into ICPC codes we used the following conditions

1. Chest pain, chest pressure, funny feeling on the chest was coded as A11
2. Limb complaints with an already proven fractures are coded as fractures

L72-L76. Otherwise they are coded in category L1 – L17

1. With multiple complaints the first one recorded is coded
2. A radial head fracture is coded as L76
3. Non-classified complaints with known diseases are classified in the purple column (other diagnoses).
4. General malaise is coded as A05
5. needle stick injury (healthcare workers) is coded as *69
6. Wounds that are sutured or glued are coded as S18
7. Pain in epigastria or stomach is coded as D02, otherwise it is D01 or D06
8. return visits due to a known fractures is coded as the fracture
9. A trauma with multiple complaints is coded as A80
10. A high impact trauma is coded as A80
11. Complaints of a limb after a trauma is coded in the L category
12. With multiple complaints within one category the first one is coded
13. Urinary catheter problems are coded as U29
14. A scheduled return visit for wound check-up or abdominal complaints are coded as *63
15. Problems with a stoma are coded as A29
16. A patient where CPR is performed is coded as K99
17. Complaints about a plaster cast is coded as A13
18. A stabbing, shouting or abuse is coded as A80
19. A transfer from another hospital as *67
20. When there was no information available, complaint is coded as *62
21. Wounds of the skin are coded as S19
22. When the complaint did not fit any category or was multi interpretable it is coded as A29
23. Flank pain not trauma related is coded as D06
24. An intoxication with medicine is coded as A84, drugs as P19 and alcohol as P16
25. When the two interpreters could not agree final coding was A29
