# Supplementary material for: Complaints and Diagnoses of Emergency Department Patients in the Netherlands: A Comparative Study of Integrated Primary and Emergency Care
Source: PLoS One. 2015 Jul 1;10(7):e0129739. doi: 10.1371/journal.pone.0129739 (PMC4488864; doi:10.1371/journal.pone.0129739)
Supplement: S2 Appendix — (DOCX) [file pone.0129739.s002.docx]

**ICD10 Codering:**

1. When no definitive diagnosis was made, the complaint is coded
2. With multiple diagnoses the first one is coded
3. A collapse due to a known cause, the cause is coded
4. A collapse with an unknown cause is coded as a collapse
5. An auto-intoxication with multiple substances is coded as X64
6. A known diabetes patient with hyperglycaemia is coded as hyperglycaemia
7. When the diagnoses said probably it was coded as not a final diagnosis
8. Injuries due to alcohol are coded as the injury
9. Alcohol abuse without injuries is coded as X65
10. When ordered back to the ED for a review, the injury is coded
11. When the diagnosis is nausea and vomiting, there is no available separate code, use R 11
12. Mild head injuries are coded as commotion cerebri S06.0
13. A high impact car mechanism without injury and no admission V99
14. Drain replacement Z46.9
15. Fever with chemotherapy is coded as R50.9
16. Left without being seen ZZ
17. The flu J11
18. Complaints of plaster Z46.9
19. General symptoms R68.8
20. High INR D68.9
21. Mild head injury with a wound S00.9
22. A trauma without an injury is coded as the trauma mechanism
23. Needle stick injury of hospital employees Z71.8
24. Multiple abnormal lab results R79.8

**Grouping**

1. Wounds: Brandwond

Amputatie

Crush

Wond

Wonden

2. Sprains and strains: vervoersongeval

Inzittende auto

Letsel pees

Letsel spier

Ruptuur

Distorsie

Contusie

Oppervlakkig

Mishandeling

Val van ladder

Gebeten

Observatie

3. Head injury: comotio

Letsel hoofd

Hersenletsel

Intracranieel

4. Fractures and dislocations: fractuur

Fracturen

Luxatie

5. Abdominal pain, unspecified: buikpijn R10.4 Pijn onderbuik R10.3

Pijn bovenbuik R10.1

6. Chestpain, unspecified: pijn op de borst R07.4

Pijn op de borst bij ademhaling R07.1

Overige pijn op de borst R07.3

7. Respiratory infections: Pneumonie J18.9

Infectie bovenste luchtweg J06.9

COPD met acute infectie J44.0

Infectie onderste luchtwegen J22

Sepsis door strepto pneumonia A40.3

Acute bronchiolitis J21.9

Acute bronchiolitis door RS virus J21.0

Influenza met overage manifestaties luchtwegen J10.1

Observatie ivm verdenking TBC Z03.0

HIV leidend tot PCP B20.6

Infeluenza met pneumonie J10.0

Pneumonie door streptococcus J13

Pneumonie door klebsiella J15.0

Pneumonie bij elders geclassificeerde aandoening J17.3

Overig pneumonie J18.8

Acute bronchitis J20/J20.9

8. Chronic respiratory disease: exc COPD J44.1

COPD J44.9

Astma J45.9

Status astmaticus J46

Astma J45

9. Acute Coronary Syndrome: myocard infarct I21.9

Instabiel AP I20.0

AP I20.9

Recidief myocard infarct I22

10. Cardiac dysrhythmias: AF I48

Total AV block I44.2

Ritmestoornis, unspec I49.9

SVT I47.1

Ventriculaire premature depolarisatie I49.3

Hartritme stoornissen I49.8

Hartstilstand I46.0/I46.9

11. Abdominal infection, unspecified: diaree verondersteld infectious A09

Enteritis door campylobacter A04.5

Intestinale virusinfectie A08.4

lymfanigits (mesenterica) I89.1

12. Specific abdominal infections: appendicitis K35.9

Divertikelziekte (diverticulitis) K57.9

Acute cholecystitis K81.0

Acute pancreatitis K85.9

Gastritis K29.7

Divertikelziekte met perforatie en abces K57.8

Cholecystitis met steen K80.4

Cholangitis K83.0

Acute pancreatitis door gal K85.1

Acute tubule-interstitiele nefritis N10

13. Specific abdominal diagnosis: GE bloeding k92.2

Cholelithiasis K80.2

Constipatie K59.0

Overigr cholelithiasis K80.8

Crohn K50.9

Ulcus ventriculi K25

Cholelithiasis K80.5

Gastro-oesofagaal laceratie-bloedingssyndroom K22.6

Ulcus duodeni K26

Ulcus pepticum K27

Ziekten van maag en duodenum K31.8

Gastroenteritis en colitis door straling K52.0

Niet infectieuze gastro-enteritis K52.9

Angiodysplasie van het colon K55.2

Paralytische ileus K56.0

Darmobstructie K56.6

Ileus K56.7

Darmperforatie K63.1

Darmziekte K63.9

Obstructie galweg K83.10

Melaena K92.1

Ascites R18

14. Infection, unspecified: Sepsis A41.9

Virus infectie B34.9

Influenza, niet geidentificeerd J11

Bacteriele infectie A49.9

Spec sepsis A41.8

Virus infectie, unspec B34

Infectie hemophilus A49.2

15. Koorts R50.9/R50.8

16. Skin infections: Herpes Zoster B02

Lokale infectie huid L08.8

Cellulitis L03.9

Lokale infectie van de huid L08.9

Impetigo L01

Huidabces L02.0

Huidabces L02.3

Huidabces L02.4

Cellulitis vinger teen L03.0

17. Infection ENT area: Otitis Media H66.9

Acute tonsillitis J03.9

Peritonsillair abces J36

Neuronitis vestibularis H81.2

Virale conjunctivitis B30.9

Acute sereuze otitis media H65.0

Acute sinusitis J01.9

Acute faryngitis J02.9

Acute tonsillitis J03

Chronische sinusitis frontalis J32.1

Periapicaal abces K04.7

Sialoadenitis K11.2

Cellulitis en abces mond K12.2

18.Vascular diseases: Longembolie I26

Flebitis en tromboflebitis I80.2

Longembolie zonder vermelding cor pulmonale I26.9

Atherosclerotische cardiovasculaire ziekte I25.0

Atherosclerose van arterien in extremiteiten I70.2

Perifere vaatziekten I73.9

Embolie bovenste extremiteit I74.2

Aandoening arterien bij elders geclassificeerde ziekte I79.8

Embolie en trombose overage venen I82.8

Hypertensie I15

Dissectie I71.0

19.Urogenital diseases: cystitis N30.0

Niersteen N20.0

Retentie blaas R33

Nierkoliek N23

Hematurie unspec R31

Uretersteen N20.1

Epididymitis N45.9

Renal en perirenaal abces N15.1

Acute nier insufficientie N17.9

Urolithiasis N20.9

Cystitis N30.9

Bloeding prostaat N42.1

Balanitis N48.1

Aandoening penis N48.9

20.Endocrine diseases: Hyperglykemie R73.9

Insulin afhankelijke DM E10

Hypoglycemie E16.2

Unspec DM E14

SIADH E22.2

Addison crise E27.2

21.Medical complications: infectie na medische verrichting T81.4

Bloeding na medische verrichting T81.0

Ongewenst gevolg geneesmiddel T88.7

Openspringen operatie wond T81.3

Complicatie medische verrichting T1.8

Vasculaire complicatie na medische verrichting T81.7

Mechainsche complicatie van dialyse catheter T82.4

Infectie als gevolg van vasculaire hulpmiddelen T82.7

Infectie agv inwendige gewrichtsprothese T84.5

Infectie algv inwendig fixatie material T84.6

Ongewenst gevolg bij therapeutisch gebruik antineoplasma Y43.3

Ongewenst gevolg bij therapeutisch gebruik overig geneesmiddel Y57.8

22.Neurological diseases: CVA I63.9

Convulsies R56.8

TIA G45.9

Intra cerebrale bloeding I61.9

Hoofdpijn R51

Epilepsie G40.9

St. epilepticus G41.9

Convulsie R56

Koortsconvulsie R56.0

Migraine G43.9

Bells palsy G51.0

Aandoening lumbosacrale wortel G54.9

SAB I60.9

Bacterial meningitis G00.9

Niet etterige meningitis G03.0

Ziekte van Parkinson G20

St. migrainosus G43.2

Transient global amnesia G45.4

Encefalopathie G93.4

Intra cerebrale bloeding in hemisfeer I61.2

Intra cerebrale bloeding in cerebellum I61.4

Dysfasie en afasie R47.0

Virale encephalitis A86

23.Periferal nerve: aandoening nervus ulnaris G56.2

Aandoening nervus radialus G56.3

Mononeuropathie bovenste extremiteit G56.8

Mononeuropathie onderste extremiteit G57.9

Polyneuropathie G62.9

Polyneuropathie bij aandoening botstelsel G63.9

Radiculopathie M54.1

Lumbago met ischias M54.4

24.Intoxication auto intox, niet gespec X64

Alcohol intox X65

Intox sedative X61

Intox drugs X62

Alcohol T51.9

Niet gespec drugs T40.6

Opzettelijk intox niet opioide X60

Opzettelijk intox geneesmiddelen X63

Opzettelijk intox chemicalien X69

25.Bijstellen hulpmiddel Z46.9

26.Syncope R55

27. Hoofdpijn R51

28. Overig HIV B24

Unspecified dementia F03

Delirium F05.9

Glaucoom H40.9

Visuele stoornissen H53.8

BPPD H81.1

Pleuravocht J91

Orthostatische hypotensie

29. Maligniteit Maligne neoplasma C78.5

Bcel lymfoom C85.1

Multipel myeloom C90.1

Anemie bij neoplasma D63.0

30. bloedziekten ijzergebreksanemie D50.0

Sikkelcel met crise D57.0

Anemie D64.9

Hemorrhagische aandoeningen D69.8

Methemoglobinemie D74.9

31. Psychiatric illnesses unsp niet organische psychose F29

Paniekstoornis F41.0

Angst stoornis F41.9

PTSS F43.1

Dissociatieve motorische stoornis F44.4

Gedragsstoornis F91.9

32. other Cardiac diseases Acute pericarditis I30.9

Aorta klep insufficiante I35.2

Aorta klep stenose en insufficientie I35.9

Hartdecompensatie I50.0/.1/.9

33. Musculoskeletal/joint

34. Gynaecological diseases

35. Cardiac complaints, non-specific

36 Respiratory complaint, unspec

37. Shortness of breath

38. Abdominal complaint, unspec

39. Neurological complaints unspec

40, traumatic injuries, other

41. Unplanned returned visits

99. Missing data
